# Phenotypic effects of the circadian gene Cryptochrome 2 on cancer-related pathways

**DOI:** 10.1186/1471-2407-10-110

**Published:** 2010-03-24

**Authors:** Aaron E Hoffman, Tongzhang Zheng, Yue Ba, Richard G Stevens, Chun-Hui Yi, Derek Leaderer, Yong Zhu

**Affiliations:** 1 Department of Epidemiology and Public Health, Yale University School of Medicine, New Haven, CT, USA; 2 Department of Community Medicine and Health Care, University of Connecticut Health Center, Farmington, CT, USA

## Abstract

**Background:**

Circadian genes continue to gain attention as important transcriptional regulators with the potential to influence a variety of biological pathways, including many cancer-related processes. The core circadian gene cryptochrome 2 (*CRY2*) is essential for proper circadian timing, and is a key component of the negative arm of the circadian feedback loop. As such, aberrant expression of *CRY2 *may influence carcinogenic processes and thereby impact cancer susceptibility.

**Methods:**

We silenced *CRY2 *in breast cancer cell lines (MCF-7) using small-interfering oligos (siRNA) and measured the impact of *CRY2 *knockdown on a number of cancer-relevant parameters. Cell cycle distribution, cell viability, and apoptotic response were measured in *CRY2 *knockdown (*CRY2*-) and normal (*CRY2*+) cell populations using flow cytometry in cells with and without exposure to a mutagen challenge. DNA damage accumulation was measured using the single cell gel electrophoresis (comet) assay, and damage was quantified using the Olive tail moment, which considers the amount and distance of DNA migration away from the nucleus, indicative of DNA strand breaks. Expression changes in cancer-relevant transcripts were measured by whole genome microarray. The Student's t-test was used for statistical comparisons, and P-values obtained from the microarray were adjusted for multiple comparisons using the false discovery rate correction, in order to obtain an adjusted Q-value for each observation.

**Results:**

The comet assay results indicated that upon exposure to the same dose of chemical mutagen, *CRY2*- cells accumulate significantly more unrepaired DNA damage than *CRY2*+ cells (P = 0.040), suggesting that *CRY2 *may be important for DNA repair. In addition, a number of transcripts with relevance for DNA damage repair displayed altered expression following *CRY2 *silencing. These included *BCCIP *(Q = 0.002), *BCL2 *(Q = 0.049), *CCND1 *(Q = 0.009), *CDKN1A *(Q < 0.001), *GADD45A *(Q = 0.002), *HERC5 *(Q < 0.001), *MCM5 *(Q = 0.042), *PPP1R15A *(Q < 0.001), *SUMO1 *(Q < 0.001), and *UBA1 *(Q = 0.023). However, no significant influence of *CRY2 *knockdown on cell cycle distributions, cell cycle checkpoints in response to mutagen challenge, or apoptotic response was detected.

**Conclusions:**

In total, these data suggest a limited, but potentially important role for *CRY2 *in the regulation of DNA damage repair and the maintenance of genomic stability. Future investigations may focus on identifying the mechanisms by which *CRY2 *may regulate the expression of transcripts with known relevance for carcinogenesis.

## Background

Although our understanding of the molecular basis for the circadian rhythm is continually evolving, the current model involves a complex interplay between environmental and endogenous factors, which include a core set of circadian genes [1]. Transcriptional and post-transcriptional interactions among these gene products results in an autoregulatory feedback system, which allows for predictable cycling of the core circadian elements [2-4]. In addition, many of the circadian genes operate as transcriptional regulators for transcripts outside of the circadian system, and recent evidence indicates that as many as 10% of all mammalian genes may be regulated to some degree by the circadian oscillatory mechanism [5-7]. As a result, disturbance of the circadian system, either through environmental exposures, or through genetic alterations in the key circadian genes, may have important implications for a variety of biological pathways.

One such core circadian gene, *CRY2*, operates in the negative arm of the circadian feedback loop as a transcriptional repressor [8]. *CRY2 *has also been shown to be involved in cancer-relevant pathways including DNA damage checkpoint control [9] and regulation of genes important for cell cycle progression [10,11]. However, *Cry1*^-/-^, *Cry2*^-/- ^transgenic mice do not display a cancer-prone phenotype in response to ionizing radiation exposure [10]. Here, we report findings from *in vitro *loss-of-function investigations into the phenotypic effects of *CRY2 *knockdown on cell cycle, apoptosis, and DNA damage response to mutagen challenge in a breast cancer cell line. We also investigate a whole genome expression array to interrogate the impact of *CRY2 *silencing on the expression of genes relevant to these pathways.

## Methods

### Cell culture and treatments

Human breast adenocarcinoma cells (MCF-7; American Type Culture Collection, Manassas, VA) were maintained in Dulbecco's modified Eagle medium (Invitrogen, Carlsbad, CA) supplemented with 10% fetal bovine serum (Invitrogen), 0.01 mg/ml bovine insulin, and 1% penicillin/streptomycin (Sigma-Aldrich, St. Louis, MO). siRNA oligos were designed and manufactured by Integrated DNA Technologies (IDT, Coralville, IA), targeting either CRY2 (Sense: 5'-UGCUUCAUUCGUUCAAUGUUAAGCCGG-3' Antisense: 5'-GGCUUAACAUUGAACGAAUGAAGCA-3') or a scrambled sequence negative control siRNA (Sense: 5'-CUUCCUCUCUUUCUCUCCCUUGUGA-3', Antisense: 5'-UCACAAGGGAGAGAAAGAGGGAAGGA-3'). Each oligo was complexed and reverse transfected using Lipofectamine RNAiMax transfection reagent (Invitrogen) at a final oligo concentration of 10 nM. Cells were either harvested 48 hours after transfection, to assay for knockdown efficiency by qPCR, or incubated with either PBS (neg. control) or 0.03% (v/v) methyl methanesulfonate (MMS, chemical mutagen) for use in subsequent assays.

### RNA isolation and quantitation

RNA samples were isolated from harvested cells using the RNA Mini Kit (Qiagen, Valencia, CA) according to the manufacturer's instructions for mammalian cells, including on-column DNA digestion. Gene expression was quantified by two-step quantitative RT-PCR, beginning with first-strand cDNA synthesis using the AffinityScript cDNA kit (Stratagene, La Jolla, CA) with oligo-dT primers, followed by quantitative real-time PCR using the Power SYBR Green PCR master mix (Applied BioSystems, Foster City, CA). The primers used for *CRY2 *amplification were: (L: ACCGGGGACTCTGTCTACTG, R: GCCTGCACTGCTCATGCT). RNA quantity was normalized using HPRT1 content, and *CRY2 *silencing was quantified prior to each treatment according to the 2^-ΔΔCt ^method. In each case, CRY2 was reduced to less than 20% of the levels seen in the mock siRNA-treated negative control (i.e. 5-fold downregulation).

### Whole genome expression microarray and pathway-based expression analysis

A whole genome expression microarray (Agilent, Inc 44k chip, performed by MoGene, LC, St Louis, MO) was used to interrogate gene expression in cells with normal and reduced *CRY2*. The results of these experiments have been uploaded to the Gene Expression Omnibus (GEO) database, and can be accessed by referencing accession #GSE14617. In order to determine whether genes involved in cell cycle regulation and DNA damage response were influenced by CRY2 knockdown, we analyzed the microarray expression data for genes in SABioscience's "Human Cell Cycle" and "Human DNA Damage Signaling Pathway" arrays (catalog numbers PAHS-020 and PAHS-029, respectively). All fold changes are the result of two biological replicates of the microarray experiment, and all observations with low intensity (<50) in both *CRY2 *normal and *CRY2 *knockdown populations have been discarded. Significantly altered genes were confirmed by qPCR, and the primers used for these reactions can be found in Additional file 1.

### Cell cycle, cell viability, and apoptosis assays

Cells with normal and reduced *CRY2 *levels were stained with propidium iodide (PI) and analyzed by flow cytometry using a fluorescence-activated cell sorter (FACS) flow cytometer (Becton Dickinson, San Jose, CA). Prior to analysis, cells were treated with either PBS or MMS for 1 hour, followed by duplicate washes and 24 hour incubation in normal growth medium. Cell populations from each treatment group were analyzed using the FlowJo flow cytometry analysis software (Tree Star, Inc., Ashland, OR), and cell phases were determined using the Watson pragmatic algorithm [12]. Cells from each of the four treatment groups (*CRY2 *+/-, MMS +/-) were also assayed for viability and apoptosis using the Vybrant Apoptosis Assay Kit #2 (Invitrogen). Briefly, cells were stained with PI and Annexin V according to the manufacturer's protocol, and scored as live, dead, apoptotic or ambiguous by flow cytometry. All results are based on the average of triplicate experiments. Raw flow cytometry images from each analysis are available in Additional file 2.

### DNA Damage Assay

DNA damage accumulation was measured by alkaline single-cell gel electrophoresis (i.e. the comet assay). Cells with normal and reduced *CRY2 *levels were incubated with MMS for one hour, followed by two PBS washes and 3 hours of recovery time. Cells were then fixed onto slides with low-melting agarose, lysed, and treated with pH>14 solution at 4°C to denature the DNA. Slides were then subjected to electrophoresis to allow damaged DNA to migrate away from the nucleus, and then stained with ethidium bromide. In order to reduce the possibility of observer bias, the prepared slides were then given to a second person who scrambled them and assigned his own arbitrary numeric label to each slide before returning them for scoring. As such, the person responsible for generating the data was unaware of the treatment status for each slide. 50 cells from each treatment group were analyzed by fluorescence microscopy using the Komet 5 comet assay analysis software. DNA damage was quantified by the software using the mean Olive tail moment calculation for each cell, as previously described [13]. Once the scoring was complete, the data were sent back to the second person who replaced the numeric label with the treatment identity. All results are from duplicate experiments performed on 50 cells each.

### Statistical Analysis

All statistical analyses were performed using the SAS statistical software (SAS Institute, Cary, NC), unless otherwise noted. *CRY2 *knockdown was assessed using the 2^-ΔΔCt ^method with RNA content normalized to the housekeeping gene *HPRT1*. Differences in cell cycle distribution were investigated by determining the percentage of cells in each phase for each treatment group. The *CRY2 *normal population was then compared to the *CRY2 *reduced population with and without mutagen challenge using the Student's t-test. Similar comparisons were made for cell viability and apoptotic response, comparing *CRY2 *normal and *CRY2 *reduced populations using the t-test. For the comet assay, comparisons were for the mean Olive tail moment in cells with reduced and normal *CRY2*, and again, the t-test was used. Due to the large number of observations present in the microarray, P-values were adjusted for multiple comparisons using the false discovery rate correction, as previously described [14], in order to obtain an adjusted Q-value for each observation.

## Results

### Silencing of CRY2 does not influence cell cycle distribution, cell cycle checkpoints, cell death, or apoptosis in response to mutagen challenge

**Figure 1:**
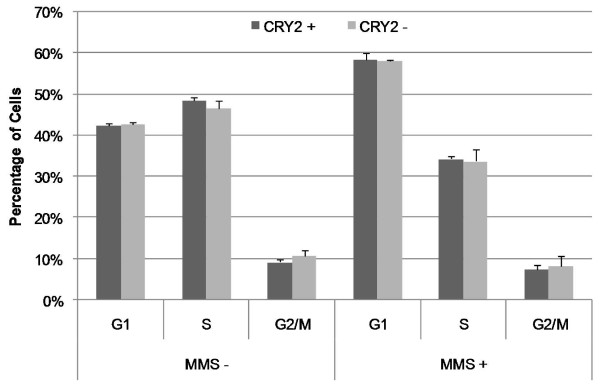
**Cell cycle distributions in *CRY2*- and *CRY2*+ cells in mock and mutagen treated cell populations**. Both in the absence and presence of mutagen, cells with reduced *CRY2 *had a similar cell cycle distribution as cells with normal *CRY2 *levels, suggesting no direct impact of *CRY2 *on cell cycle progression or cell cycle checkpoints in response to DNA damage. All results are based on the average of triplicate experiments, and error bars are for SEM.

Cell cycle distributions in mock-treated or mutagen challenged cells were measured in populations with normal (*CRY2*+) and reduced *CRY2 *levels (*CRY2*-). In both treatment groups, similar distributions were observed for *CRY2*+ and *CRY2*- cell populations, indicating that reduction of *CRY2 *alone is not sufficient to significantly impact cell cycle regulation. The cell phase distributions in mock treated cells were: *CRY2*+: G1, 42.3%, S, 48.5%, G2/M, 9.2%; *CRY2*-: G1, 42.7%, S, 46.6%, G2/M, 10.8% (Figure 1). In addition, a similar magnitude of G1 delay was observed in both the *CRY2*+ and *CRY2*- populations following mutagen challenge, suggesting that *CRY2 *does not significantly influence DNA damage-induced cell cycle checkpoint control. Cell cycle distributions in mutagen treated cells were: *CRY2*+: G1, 58.3%, S, 34.3%, G2/M, 7.4%; CRY2-: G1, 58.1%, S, 33.7%, G2/M, 8.2%.

**Figure 2:**
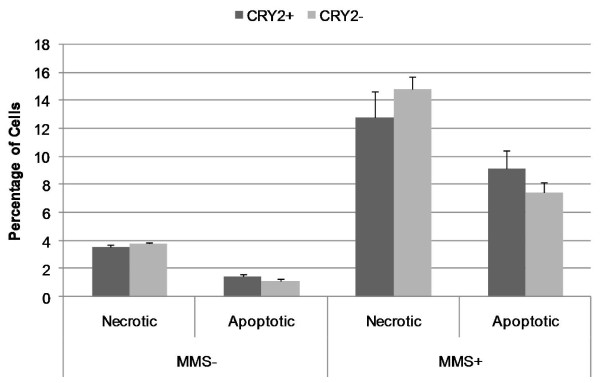
**Cell death and apoptosis in *CRY2*+ and *CRY2*- cell populations**. No differences were observed in *CRY2 *knockdown cells in terms of necrosis or apoptosis, with or without mutagen exposure. All results are based on the average of triplicate experiments, and error bars are for SEM.

Flow cytometric analyses of necrosis and apoptosis in mock and mutagen treated cells also revealed no significant differences in *CRY2*+ and *CRY2*- cell populations. Both populations had similarly low percentages of necrotic and apoptotic cells in the mock treated populations: *CRY2*+: necrotic 3.5%, apoptotic, 1.5%; *CRY2*-: necrotic, 3.8%, apoptotic 1.2% (Figure 2). The percentage of necrotic and apoptotic cells in both these populations increased by similar margins following mutagen challenge: *CRY2*+: necrotic 12.8%, apoptotic, 9.1%; *CRY2*-: necrotic, 14.8%, apoptotic 7.5%. No comparisons, either in the percentage of necrotic or apoptotic cells, or in the magnitude of the response following mutagen challenge, were statistically significant.

### CRY2 knockdown results in increased accumulation of mutagen-induced DNA damage

**Figure 3:**
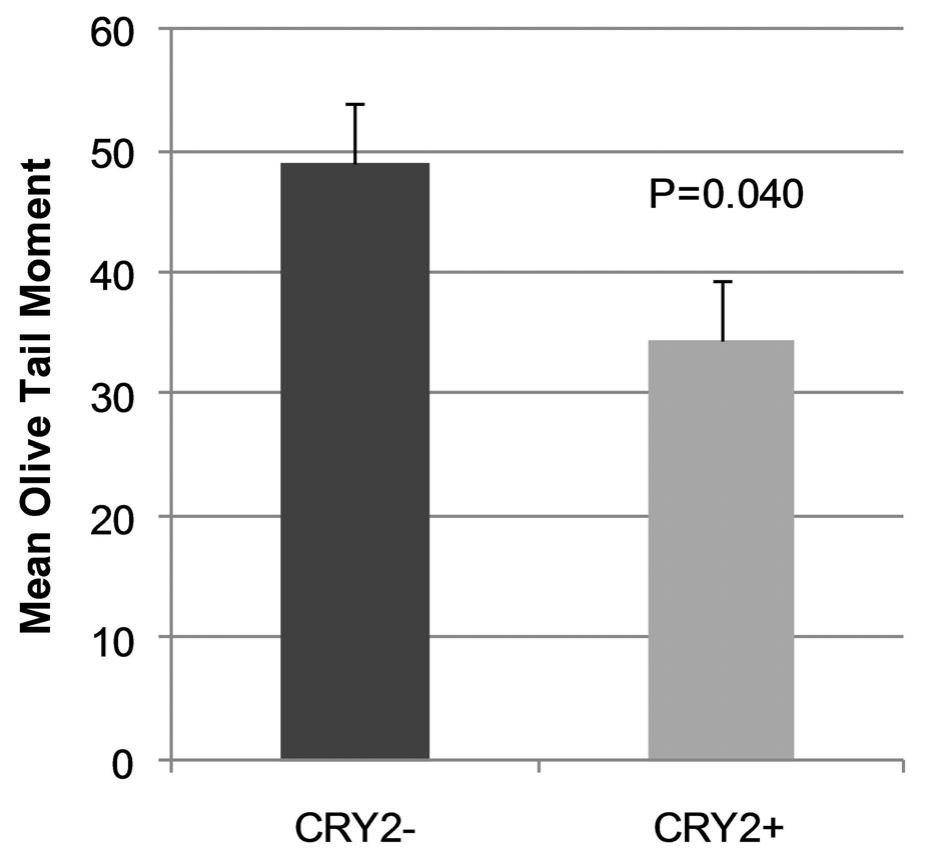
**Comet assay results for cells with reduced and normal *CRY2***. DNA damage was measured by the Olive tail moment, which considers the amount and distance of DNA migration away from the nucleus, indicative of DNA strand breaks. Higher values correspond to increased damage. Upon exposure to the same concentration of mutagen, *CRY2*- cells accumulated significantly more DNA damage compared to normal cells (P = 0.040). Comet assay results are from duplicate experiments performed on 50 cells each. All data are aggregated (i.e. 100 cells per treatment), and error bars are for SEM.

The single cell gel electrophoresis (comet) assay was used to evaluate the extent of DNA damage following mutagen challenge in *CRY2*+ and *CRY*-populations. DNA damage was quantified using the Olive tail moment measure, which incorporates the distance of DNA migration from the nucleus, as well as the percentage of total DNA which has migrated away from the nuclear core, indicating that it has been relaxed or broken. The mean Olive tail moment in *CRY2*- cells was significantly higher than that in CRY2+ cells (P = 0.04), indicating that the same levels of mutagen exposure result in greater DNA damage in cells with reduced *CRY2 *(Figure 3). This is especially notable given that *CRY2*- cells do not exhibit decreased survival or increased apoptosis, suggesting that they survive equally well despite the increased level of DNA damage; a phenotype which could lead to tumor promotion.

### *CRY2 *knockdown results in expression changes in genes in the DNA damage response and cell cycle regulatory pathways

A whole genome expression microarray was performed using RNA isolated from *CRY2*+ and *CRY2*- cell populations. Using the genes in the SABioscience cell cycle and DNA damage response arrays, we identified 10 genes in these pathways which displayed significantly altered expression following *CRY2 *knockdown (Q-value < 0.05). Expression of each of these genes was also determined by qPCR for each cell treatment in order to confirm the array results. All genes were altered in the same direction when measured by array or qPCR, and all fold changes were of similar magnitude, with the exception of *SUMO1*, which was two-fold upregulated in the array, but only 1.32 upregulated when measured by qPCR (Table 1). Among the significantly altered gene set were cyclin dependent kinase inhibitor p21 (*CDKN1A*; fold change = 1.7, Q < 0.001) along with its interacting protein (*BCCIP*; fold change = 2.3, Q = 0.002). Perhaps most interesting, however, was the induction of cyclin D1 (*CCND1*; fold change = 1.5, Q = 0.009) in cells with reduced *CRY2*. *CCND1 *is a firmly established oncogene that is often overexpressed in primary breast cancers [15], and while aberrant overexpression of *CCND1 *is often due to gene amplification, up to 50% of breast cancers display increases in *CCND1*, many of which cannot be explained by copy number variations, indicating that alternative mechanisms such as transcriptional dysregulation must be involved [16].

**Table 1:** Cell cycle and DNA damage repair genes with altered expression following *CRY2 *silencing.

Gene	RefSeq	Description	Array Fold Change	Q-Value	qPCR Fold Change
BCCIP	NM_078469	BRCA2 and CDKN1A interacting protein	2.33	0.0021	1.57
BCL2	NM_000633	B-cell CLL/lymphoma 2	-1.47	0.0487	-1.50
CCND1	NM_053056	Cyclin D1	1.51	0.0087	1.33
CDKN1A	NM_000389	Cyclin-dependent kinase inhibitor 1A (p21, Cip1)	1.71	0.0001	1.98
GADD45A	NM_001924	Growth arrest and DNA-damage-inducible, alpha	1.92	0.0015	2.16
HERC5	NM_016323	Hect domain and RLD 5	3.93	0.0000	2.28
MCM5	NM_006739	Minichromosome maintenance complex component 5	-1.58	0.0415	-1.46
PPP1R15A	NM_014330	Protein phosphatase 1, regulatory (inhibitor) subunit 15A	2.55	0.0004	2.45
SUMO1	NM_001005781	SMT3 suppressor of mif two 3 homolog 1 (S. cerevisiae)	2.00	0.0002	1.32
UBA1	NM_003334	Ubiquitin-like modifier activating enzyme 1	1.58	0.0232	1.43

## Discussion

*CRY2*, in conjunction with *CRY1 *and the period genes, (*PER1*, *PER2*, and *PER3*) operates on the negative arm of the circadian system and is essential for maintaining proper circadian rhythm [17]. However, due to the complex nature of circadian gene interactions, which include pre- and post-transcriptional regulation, it remains difficult to determine the direct phenotypic impact of a single gene, especially in light of the potential for overlapping functions and compensatory mechanisms among the core circadian proteins. Nevertheless, the observation that cells with reduced *CRY2 *accumulate greater DNA damage is consist with the general understanding that circadian genes may directly influence organismal susceptibility to genotoxic stress [18].

The finding that *CCND1 *is induced following *CRY2 *knockdown, while not proof of direct inhibition of *CCND1 *by *CRY2*, does provide the intriguing possibility that the aberrant overexpression of *CCND1 *observed in several cancer types could be, in part, the result of circadian-mediated transcriptional dysregulation. Evidence for this association was provided by an earlier study which showed that enforced expression of *PER2 *resulted in a 56% reduction in *CCND1 *levels *in vitro *[19]. Interestingly, in addition to the positive regulator of cell cycle progression, *CCND1*, a crucial cyclin-dependent kinase inhibitor, *CDKN1A *(also known as *P21*), was also induced after *CRY2 *knockdown. This finding is consistent with a recent report demonstrating that clock-deficient mice have increased levels of p21, resulting in decreased cellular proliferation rates [20]. That both *CCND1 *and *CDKN1A*, which influence cell cycle progression in opposite directions, were each upregulated after *CRY2 *knockdown provides a potential explanation for the lack of observable phenotypic impact of *CRY2 *silencing on cell cycle distributions. Given the importance of these cell cycle regulators in a variety of cancer types, additional exploration into the nature of these associations is warranted.

Despite evidence that cryptochromes may be involved in cancer-associated processes [11], two *in vivo *studies did not find a cancer-prone phenotype in double mutant mice lacking both crytpochrome genes (i.e. *Cry1*-/-*Cry2*-/-). For example, *Cry1*-/-*Cry2*-/- mice did not have poorer survival rates than wild type (WT) mice following exposure to ionizing radiation [10]. In addition, fibroblasts derived from these mice did not have deficient DNA damage capacities compared to those derived from wild type mice, and cell cycle checkpoints were similarly unaffected. Another study of *Cry1*-/-*Cry2*-/- also showed that while these mice were significantly smaller than their WT counterparts, they did not have any obvious malignancies, and they remained reproductively fit [21]. Interestingly, in mice which are predisposed to cancer due to a mutation in p53, addition of the *Cry *mutation results in sensitization of p53 mutant cells to apoptosis and thus decreased cancer risk and increased survival [22]. In addition, there is a strong circadian rhythm in nucleotide excision repair activity in the mouse brain, caused at least partly by circadian regulation of xeroderma pigmentosum A (XPA) [23]. Cry negatively regulates this activity, and fibroblasts from *Cry1*-/-*Cry2*-/- mice exhibit 3-fold induction of XPA protein. It should be noted, however, that each of these studies employed double mutant mice, and are thus not necessarily reflective of the condition which may exist in the absence of *Cry2 *only. In fact, a behavioral and molecular study of the effect of cryptochromes on light entrainment and circadian regulation showed very different phenotypes for *Cry1*-/-*Cry2*-/- mice compared to mice lacking *Cry1 *only [24], and an earlier study of *Cry*-/- mice suggested that reductions in either cryptochrome alone have effects which are directly opposed to one another [8]. To the best of our knowledge, no study has yet explored the response of *Cry2*-/- only to mutagen challenge, or the effect of induced degradation of *CRY2 *on DNA repair. It should also be noted that our data are generated using cells with wild-type p53. Thus, future studies may wish to investigate the effect of *CRY2 *knockdown in mutant p53 cells, especially in light of the recent evidence suggesting differential effects of *Cry *mutations against the p53 mutant background, as outlined above.

While changes were detected in DNA damage accumulation following *CRY2 *knockdown, no differences were observed in other cancer-related pathways. However, previous studies have shown that induction of the DNA damage response pathway is an important early event in determining whether precursor lesions will develop into malignancies [25], and those authors suggest that mutations which disrupt the DNA damage response may allow tumor progression. In another study, Gorgoulis *et al. *demonstrate that a DNA damage response is present in precancerous lesions, also suggesting that disruption of this pathway could be an important determinant of progression to carcinoma [26]. One interesting aspect of our phenotypic assays was the increase in DNA damage observed in *CRY2*- cell populations in the absence of decreased survival or increased apoptosis. If in fact reduced *CRY2 *results in increased DNA damage without triggering increases in cell death or apoptosis, this could potentially lead to cancer, as damaged cells could survive and be allowed to proliferate. As such, this phenotype warrants further investigation.

## Conclusion

In summary, these data suggest a limited, but potentially important role for *CRY2 *in maintaining genomic stability. Future investigations may wish to focus on the transcriptional influence of *CRY2 *on oncogenic *CCND1*, and the relationship with *CDKN1A*, as these findings have the potential for broad impact on a number of cancer types. In addition, given the evidence that response to cancer therapy may be influenced by circadian cycling [18,27,28], and the fact that *CRY2 *may influence the accumulation of DNA damage, future investigations into the effects of *CRY2 *on response to treatment are also warranted.

## Competing interests

The authors declare that they have no competing interests.

## Authors' contributions

AEH carried out the knockdown experiments, as well as the cell cycle and apoptosis studies and the analysis of the array data, and drafted the manuscript. YB, CY, and DL each participated in the comet assay. RS and TZ participated in the design of the study. YZ conceived the study, and participated in its design and coordination. All authors read and approved the final manuscript.

## Pre-publication history

The pre-publication history for this paper can be accessed here:

http://www.biomedcentral.com/1471-2407/10/110/prepub

## Supplementary Material

Additional file 1 Primer sequences used for qPCR confirmation of genes identified as significantly altered by microarray. Click here for file

Additional file 2 Flow cytometry images from the cell cycle (2A) and cell viability/apoptosis (2B) analyses. Click here for file

## References

[B1] Oster H The genetic basis of circadian behavior Genes Brain Behav 2006 5 Suppl 2 73 79 16681802 10.1111/j.1601-183X.2006.00226.x

[B2] Young MW Kay SA Time zones: a comparative genetics of circadian clocks Nat Rev Genet 2001 2 9 702 715 10.1038/35088576 11533719

[B3] Reppert SM Weaver DR Molecular analysis of mammalian circadian rhythms Annu Rev Physiol 2001 63 647 676 10.1146/annurev.physiol.63.1.647 11181971

[B4] Reppert SM Weaver DR Coordination of circadian timing in mammals Nature 2002 418 6901 935 941 10.1038/nature00965 12198538

[B5] Le Minh N Damiola F Tronche F Schutz G Schibler U Glucocorticoid hormones inhibit food-induced phase-shifting of peripheral circadian oscillators Embo J 2001 20 24 7128 7136 10.1093/emboj/20.24.7128 11742989 PMC125339

[B6] Storch KF Lipan O Leykin I Viswanathan N Davis FC Wong WH Weitz CJ Extensive and divergent circadian gene expression in liver and heart Nature 2002 417 6884 78 83 10.1038/nature744 11967526

[B7] Duffield GE Best JD Meurers BH Bittner A Loros JJ Dunlap JC Circadian programs of transcriptional activation, signaling, and protein turnover revealed by microarray analysis of mammalian cells Curr Biol 2002 12 7 551 557 10.1016/S0960-9822(02)00765-0 11937023

[B8] Thresher RJ Vitaterna MH Miyamoto Y Kazantsev A Hsu DS Petit C Selby CP Dawut L Smithies O Takahashi JS Sancar A Role of mouse cryptochrome blue-light photoreceptor in circadian photoresponses Science 1998 282 5393 1490 1494 10.1126/science.282.5393.1490 9822380

[B9] Unsal-Kacmaz K Mullen TE Kaufmann WK Sancar A Coupling of human circadian and cell cycles by the timeless protein Mol Cell Biol 2005 25 8 3109 3116 10.1128/MCB.25.8.3109-3116.2005 15798197 PMC1069621

[B10] Gauger MA Sancar A Cryptochrome, circadian cycle, cell cycle checkpoints, and cancer Cancer Res 2005 65 15 6828 6834 10.1158/0008-5472.CAN-05-1119 16061665

[B11] Matsuo T Yamaguchi S Mitsui S Emi A Shimoda F Okamura H Control mechanism of the circadian clock for timing of cell division in vivo Science 2003 302 5643 255 259 10.1126/science.1086271 12934012

[B12] Watson JV Chambers SH Smith PJ A pragmatic approach to the analysis of DNA histograms with a definable G1 peak Cytometry 1987 8 1 1 8 10.1002/cyto.990080101 3803091

[B13] Olive PL Banath JP Durand RE Heterogeneity in radiation-induced DNA damage and repair in tumor and normal cells measured using the "comet" assay Radiat Res 1990 122 1 86 94 10.2307/3577587 2320728

[B14] Benjamini Y Hochberg Y Controlling the False Discovery Rate: A Practical and Powerful Approach to Multiple Testing Journal of the Royal Statistical Society Series B (Methodological) 1995 57 1 289 300

[B15] Buckley MF Sweeney KJ Hamilton JA Sini RL Manning DL Nicholson RI deFazio A Watts CK Musgrove EA Sutherland RL Expression and amplification of cyclin genes in human breast cancer Oncogene 1993 8 8 2127 2133 8336939

[B16] Arnold A Papanikolaou A Cyclin D1 in breast cancer pathogenesis J Clin Oncol 2005 23 18 4215 4224 10.1200/JCO.2005.05.064 15961768

[B17] Kume K Zylka MJ Sriram S Shearman LP Weaver DR Jin X Maywood ES Hastings MH Reppert SM mCRY1 and mCRY2 are essential components of the negative limb of the circadian clock feedback loop Cell 1999 98 2 193 205 10.1016/S0092-8674(00)81014-4 10428031

[B18] Antoch MP Kondratov RV Takahashi JS Circadian clock genes as modulators of sensitivity to genotoxic stress Cell Cycle 2005 4 7 901 907 15917646 10.4161/cc.4.7.1792 PMC3774065

[B19] Xiang S Coffelt SB Mao L Yuan L Cheng Q Hill SM Period-2: a tumor suppressor gene in breast cancer J Circadian Rhythms 2008 6 4 10.1186/1740-3391-6-4 18334030 PMC2365929

[B20] Grechez-Cassiau A Rayet B Guillaumond F Teboul M Delaunay F The circadian clock component BMAL1 is a critical regulator of p21WAF1/CIP1 expression and hepatocyte proliferation J Biol Chem 2008 283 8 4535 4542 10.1074/jbc.M705576200 18086663

[B21] Kondratov RV Gorbacheva VY Antoch MP The role of mammalian circadian proteins in normal physiology and genotoxic stress responses Curr Top Dev Biol 2007 78 173 216 full_text 17338917 10.1016/S0070-2153(06)78005-X

[B22] Ozturk N Lee JH Gaddameedhi S Sancar A Loss of cryptochrome reduces cancer risk in p53 mutant mice Proc Natl Acad Sci USA 2009 106 8 2841 2846 10.1073/pnas.0813028106 19188586 PMC2634797

[B23] Kang TH Reardon JT Kemp M Sancar A Circadian oscillation of nucleotide excision repair in mammalian brain Proc Natl Acad Sci USA 2009 106 8 2864 2867 10.1073/pnas.0812638106 19164551 PMC2629438

[B24] Vitaterna MH Selby CP Todo T Niwa H Thompson C Fruechte EM Hitomi K Thresher RJ Ishikawa T Miyazaki J Takahashi JS Sancar A Differential regulation of mammalian period genes and circadian rhythmicity by cryptochromes 1 and 2 Proc Natl Acad Sci USA 1999 96 21 12114 12119 10.1073/pnas.96.21.12114 10518585 PMC18421

[B25] Bartkova J Horejsi Z Koed K Kramer A Tort F Zieger K Guldberg P Sehested M Nesland JM Lukas C Ørntoft T Lukas J Bartek J DNA damage response as a candidate anti-cancer barrier in early human tumorigenesis Nature 2005 434 7035 864 870 10.1038/nature03482 15829956

[B26] Gorgoulis VG Vassiliou LV Karakaidos P Zacharatos P Kotsinas A Liloglou T Venere M Ditullio RA Jr Kastrinakis NG Levy B Kletsas D Yoneta A Herlyn M Kittas C Halazonetis TD Activation of the DNA damage checkpoint and genomic instability in human precancerous lesions Nature 2005 434 7035 907 913 10.1038/nature03485 15829965

[B27] Gorbacheva VY Kondratov RV Zhang R Cherukuri S Gudkov AV Takahashi JS Antoch MP Circadian sensitivity to the chemotherapeutic agent cyclophosphamide depends on the functional status of the CLOCK/BMAL1 transactivation complex Proc Natl Acad Sci USA 2005 102 9 3407 3412 10.1073/pnas.0409897102 15689397 PMC546637

[B28] Lis CG Grutsch JF Wood P You M Rich I Hrushesky WJ Circadian timing in cancer treatment: the biological foundation for an integrative approach Integr Cancer Ther 2003 2 2 105 111 10.1177/1534735403002002002 15035897

