# Supplementary material for: Phenotypic effects of the circadian gene Cryptochrome 2 on cancer-related pathways
Source: BMC Cancer. 2010 Mar 24;10:110. doi: 10.1186/1471-2407-10-110 (PMC2860360; doi:10.1186/1471-2407-10-110)
Supplement: Additional file 1 — Primer sequences used for qPCR confirmation of genes identified as significantly altered by microarray. [file 1471-2407-10-110-S1.DOC]

**Additional file 1**. Primer sequences used for qPCR confirmation of genes identified as significantly altered by microarray.

|  |  |  |
| --- | --- | --- |
| Gene | Left Primer | Right Primer |
| BCCIP | tgcagcaGctttttctaaagg | ggcttatgaaaccaaaaacctc |
| BCL2 | gataacggaggctgGgatg | agcctgcagctttgtttcat |
| CCND1 | acctggatgctggaGgtct | ggggatggtctccttcatct |
| CDKN1A | gcagaccagcatgacaGAtt | gccagggtatgtacatgagga |
| GADD45A | gcctgtgagtgagtgcagaa | ccttatccatcCtttcggtct |
| HERC5 | tgtGgacgcttcagaaaatg | gctgccgacctaagataagc |
| MCM5 | gacttcatgcccaccatctt | ccacacttcActcggcagta |
| PPP1R15A | gaggaagaggaagctgctga | cacCtttctggcctttaggg |
| SUMO1 | ccaaaagaActgggaatgga | caattccgttttgaacacca |
| UBA1 | tactcccggcagcTgtatgt | gccctggtcatgtagggtaa |
