# Supplementary material for: Phenotypic effects of the circadian gene Cryptochrome 2 on cancer-related pathways
Source: BMC Cancer. 2010 Mar 24;10:110. doi: 10.1186/1471-2407-10-110 (PMC2860360; doi:10.1186/1471-2407-10-110)

**Additional file 2A:** Cell cycle FACS images


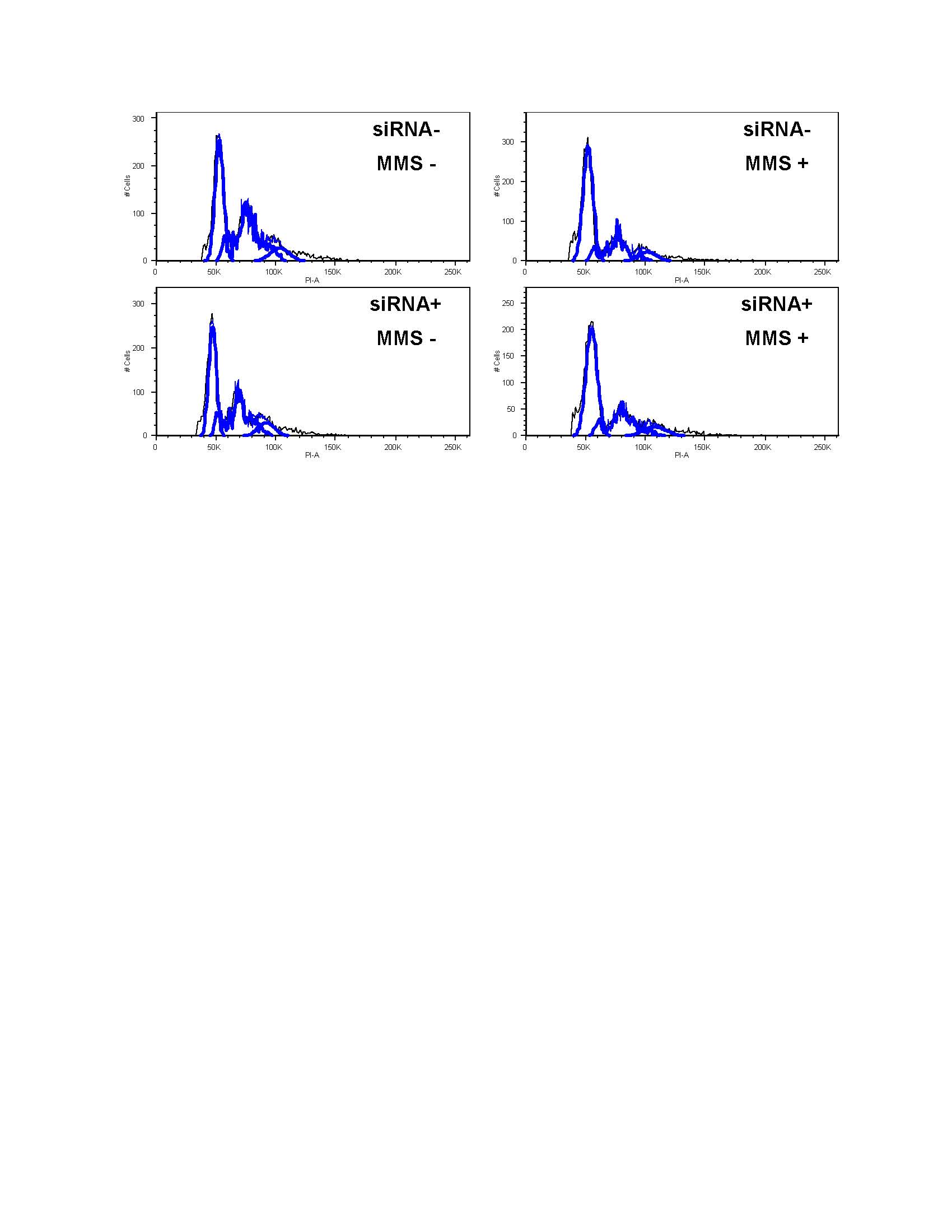


**Additional file 2B:** FACS Images for cell viability and apoptosis. Treatments are: A) *CRY2*+/MMS-, B) *CRY2*+/MMS+, C) *CRY2*-/MMS-, D) *CRY2*-/MMS+


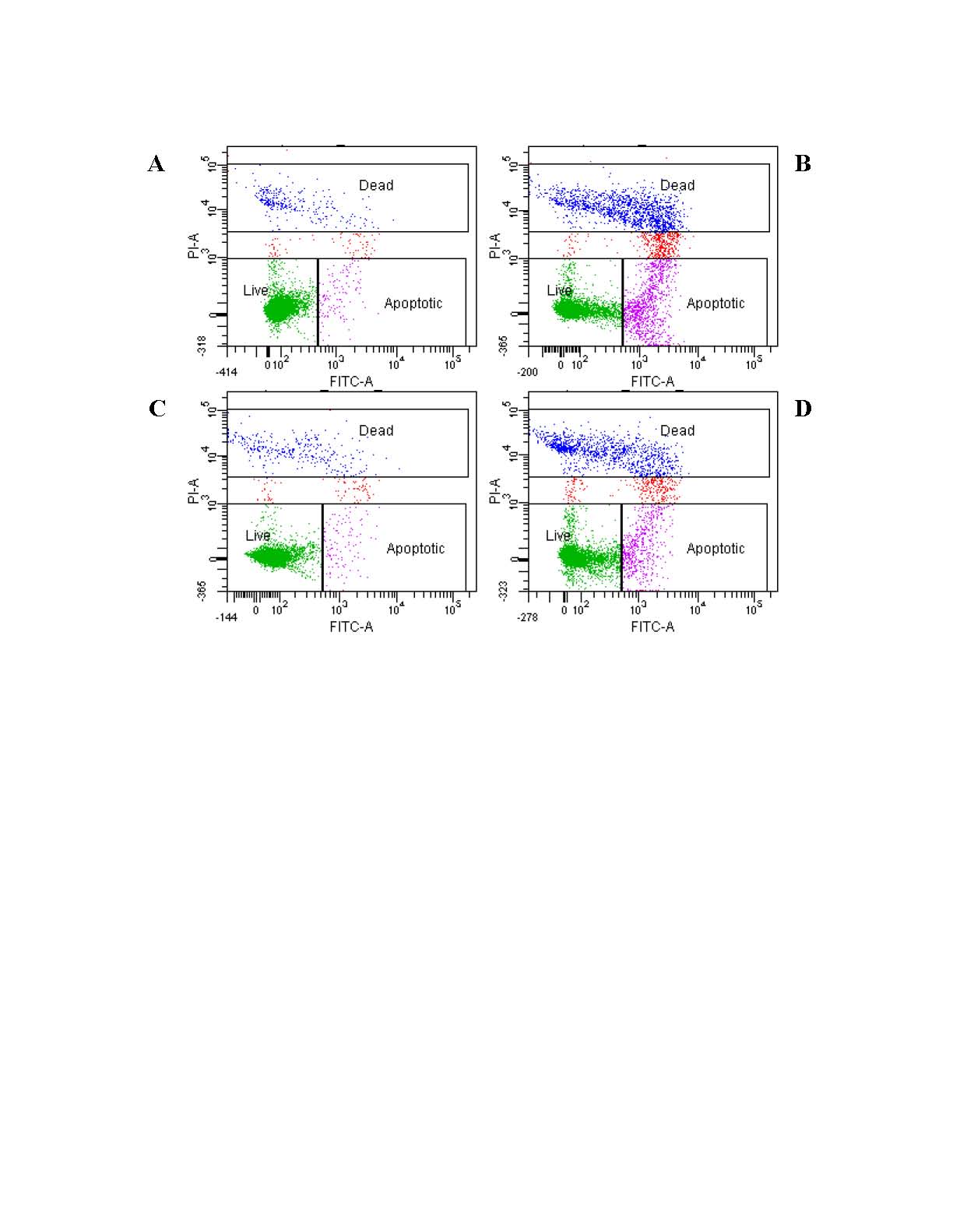

Supplement: Additional file 2 — Flow cytometry images from the cell cycle (2A) and cell viability/apoptosis (2B) analyses. [file 1471-2407-10-110-S2.DOC]
